# Immunological Compatibility of Bone Tissues from Alpha-1,3-galactosyltransferase Knockout Pig for Xenotransplantation

**DOI:** 10.1155/2018/1597531

**Published:** 2018-06-05

**Authors:** Se Eun Kim, Kyung Won Kang, Suna Gu, Seongsoo Hwang, Sun A Ock, Kyung Mi Shim, Kwangsik Jang, Seok Hwa Choi, Sang-Myeong Lee, Seong Soo Kang

**Affiliations:** ^1^College of Veterinary Medicine, BK21 Plus Project Team and Biomaterial R&BD Center, Chonnam National University, 77 Yongbong-ro, Buk-gu, Gwangju 61186, Republic of Korea; ^2^Division of Biotechnology, College of Environmental and Bioresource Sciences, Chonbuk National University, 79 Gobong-ro, Iksan-si, Jeollabuk-do 54596, Republic of Korea; ^3^Animal Biotechnology Division, National Institute of Animal Science, RDA, 1500 Kongjwipatjwi-ro, Iseo-myeon, Wanju-Gun, Jeollabuk-do 55365, Republic of Korea; ^4^College of Veterinary Medicine, Chungbuk National University, 1 Chungdae-ro, Seowon-Gu, Cheongju Chungbuk 28644, Republic of Korea; ^5^Division of Biotechnology, Advanced Institute of Environment and Bioscience, College of Environmental and Bioresource Sciences, Chonbuk National University, Iksan, Jeonbuk 54596, Republic of Korea

## Abstract

We investigated whether the lack of galactosyltransferase (*α*-Gal) expression in bone tissue is associated with reduced immune response of human peripheral blood mononuclear cells (PBMCs) against pig bone tissue. When human PBMC obtained from heparinized blood of healthy volunteers was stimulated with bone extracts of pigs with *α*-1,3-galactosyltransferase knock out (*α*-Gal KO), the proliferation of human PBMCs and production of proinflammatory cytokines such as TNF-*α* and IL-1*β* were significantly reduced compared to those stimulated with bone extracts of wild type (WT) pigs. In addition, activation of CD4^+^ helper T cells and production of IL-2, IFN-*γ*, and IL-17 were reduced upon stimulation with bone tissue extracts from *α*-Gal KO pigs. This is possibly due to the lowered activities of the NF-*κ*B, p38, ERK, and JNK signaling pathways. Our findings can be used to evaluate the compatibility of bone tissues from *α*-Gal KO pigs with human bone grafting as novel natural biomaterials, thereby increasing the feasibility of future clinical applications.

## 1. Introduction

Organ transplantation is the sole treatment option available for end-stage diseases in organs such as the liver, lung, kidney, and heart [1–3]. While the demand for organ transplantation has increased rapidly, human donor organs are still in short supply. This disparity in the supply and demand for organs has increased the waiting time of patients with end-stage organ failure for organ transplantation worldwide [1, 4, 5]. Recently, interest in the transplantation of organs from other mammals (xenotransplantation) has increased to circumvent the problems associated with shortage of human organs. Currently, xenotransplantation is the only available solution to the problem of chronic organ shortage [1, 2, 5]. In particular, pigs are considered an appropriate source for xenografts because of the similarities in physiology and size between human and pig organs [6–8].

However, the main problem with xenotransplantation is the immune rejection of the pig organ grafts, which is mediated primarily by the natural human anti-Gal antibodies to the *α*-Gal epitope (Gal*α*1-3Gal*β*1-4G1cNAc-R) expressed on porcine cells. The *α*-Gal epitope is a common carbohydrate structure in most mammalian species including pigs, but not in humans, apes, and the old world monkeys. Therefore, when a pig organ is transplanted into humans, hyperacute rejection (HAR) is triggered with the binding of human *α*-gal-specific natural antibodies to the *α*-Gal epitope on pig cells, followed by complement activation [6–10].

Prevention of pig xenograft rejection in humans can be achieved by knocking out the gene encoding alpha-1,3-galactosyltransferase in pigs [6]. The alpha-1,3-galactosyltransferase knockout (*α*-Gal KO) pigs lack the *α*-Gal epitopes, do not elicit immune rejection, and are therefore compatible for xenotransplantation [11].

In particular, immunogenic protein components in the bone matrix are scarce compared to that in vascularized organs such as kidney, heart, and liver. Moreover, freeze drying, demineralized freeze drying, or gamma irradiation can be used to reduce the antigenicity of bone graft [12–15]. In practice, bone tissue is the second common transplanted biological material following blood [15]. Therefore, bone tissues from *α*-Gal KO pigs could be better candidates compared to soft tissues for xenotransplantation in humans.

Although several studies have investigated the xenotransplantation of *α*-Gal knockout pig organs such as the heart, thymus, and kidney [16–18], pig bone xenotransplantation has not been well studied. In this study, we investigated whether lack of *α*-Gal expression in bone tissue is associated with reduced inflammatory cytokine production by human peripheral blood mononuclear cells (PBMCs) and evaluated the possibility of *α*-Gal KO pig bone xenografts as an alternative to autografts and allografts.

## 2. Materials and Methods

### 2.1. Sample Preparation

**Figure 1:**
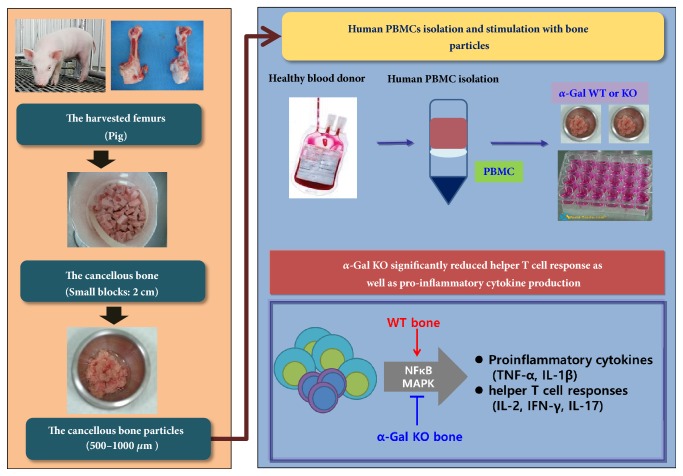
Schematic illustration of the flow sheet for the evaluation of the clinical applicability of bone tissues from alpha-1,3-galactosyltransferase knockout pig (PBMC: peripheral blood mononuclear cell, *α*-Gal KO: *α*-1,3-galactosyltransferase knock out pig, and WT: wild type pig).

In this study, fresh bones were obtained* via* lateral approach from both femurs of euthanized five homozygous *α*-Gal KO male pigs about 4–6 weeks old (Massachusetts General Hospital miniature pigs; [19, 20]) and five Yorkshire Large White pigs about 4 weeks old. Homozygous *α*-Gal KO male pigs and Yorkshire Large White pigs were supported by the National Institute of Animal Science, Rural Development Administration (RDA), Korea. The deficiency of Gal in KO pigs was confirmed as previously described [20]. The flowchart used in this study is shown in Figure 1: fresh cancellous bones were harvested aseptically from proximal femurs to ensure sufficient supply of bones for the experiments and cut into small blocks of 2 cm or less in size. The cancellous bones were washed with 70% ethanol solution and normal saline. And then, the cancellous bones were milled into particles of 500–1000 *μ*m using a bone mill and stored at −70°C.

### 2.2. Human PBMC Isolation and Stimulation with Pig Bone Extract

This study was performed with the approval of the Institutional Review Board of the Chonnam National University (1040198-140418-BR-021-02). PBMCs were obtained from heparinized blood of six healthy volunteers using Histopaque (Sigma, St. Louis, MO, USA) gradient centrifugation. Mononuclear cells were resuspended in Roswell Park Memorial Institute (RPMI-) 1640 (Gibco, Paisley, UK) and medium supplemented with 10% autologous serum and gentamicin (40 g/mL). Ground bone particles were resuspended in serum-free RPMI-1640 and passed through a cell strainer with 40 *μ*m pore size. Then, protein concentration was measured using the Bradford assay. Bone mixtures were added to PBMC cultures at a concentration of 100 *μ*g/mL. The cells were incubated at 37°C with 5% CO_2_ for 24 h for RNA extraction and for 48 h for enzyme-linked immunosorbent assay (ELISA).

### 2.3. ELISA

ELISA (Ebioscience, San Diego, CA, USA) was used to determine cytokine concentration in cell culture supernatants (TNF- *α* and IL-1*β*) after stimulating PBMCs for 48 h with bone suspension, according to the manufacturer's procedure.

### 2.4. PBMC Proliferation

PBMC proliferation was determined using dilutions of 5,6-carboxyfluorescein diacetate, succinimidyl ester (CFSE, eBioscience). Briefly, single cell suspensions were washed twice with phosphate buffered saline (PBS) and resuspended in prewarmed PBS at a cell density of 5–10 × 10^6^, followed by addition of 0.2 *μ*L 5 mM CFSE to the cell suspension. After 30 min at room temperature in the dark, 4-5 volumes of cold complete media was added to stop labeling and the cells were incubated on ice for 5 min. CFSE-labeled PBMCs were washed thrice with complete media and were stimulated for 72 h with bone extracts from WT or *α*-Gal KO pigs. PHA (5 *μ*g/mL) was used as a positive control. Cells were harvested and washed twice with PBS. Propidium iodide was added to the cell suspension and the cells were analyzed using an Accuri Flow Cytometer (BD). A minimum of 100,000 events were collected.

### 2.5. T Cell Activation Markers

PBMCs were activated with either bone extracts (WT or KO) or PHA (5 *μ*g/mL) for 24 h and harvested. After washing twice with PBS, cells were resuspended in FACS buffer, followed by staining with antibodies against CD3, CD4, CD25, and CD69 on ice for 30 min. Then, the cells were washed and resuspended in FACS buffer. The cells were analyzed for cell surface expression of CD25 or CD69 on CD3^+^CD4^+^ T cells using the Accuri Flow Cytometer.

### 2.6. Western Blotting

PBMCs were rinsed in cold PBS and lysed in RIPA buffer containing 50 mM Tris–HCl pH 7.4, 150 mM NaCl, 1 mM EDTA, 1 mM PMSF, 1% Nonidet P-40, 0.25% sodium deoxycholate, 1 mM NaF, and 1 mM Na_3_VO_4_ (all other products from Sigma Aldrich, USA) and the Complete™ Mini-EDTA-Free Protease Inhibitor Cocktail (Roche Diagnostics). After centrifugation (15 min, 12,000 × g, 4°C), the protein concentration was measured using the Bradford assay (Pierce). Total protein (50 *μ*g) was subjected to 10% sodium dodecyl sulfate-polyacrylamide gel electrophoresis (SDS-PAGE) and transferred onto polyvinylidenedifluoride (PVDF) membrane. The membrane was blocked with 5% nonfat dry milk dissolved in trisbuffered saline containing 0.05% Tween 20 (TBST) for 1 h at room temperature and probed with specific primary antibodies in 5% BSA plus TBST at 4°C overnight. The next day, the membrane was treated with horse radish peroxidase-conjugated immunoglobulin G (IgG) for 1 h at room temperature. Chemiluminescence was detected using the Pierce enhanced chemiluminescence (ECL) western blotting substrate (Thermo Scientific, Rockford, IL, USA) and visualized using LAS4000 mini imaging system (Fuji photo film, Japan).

### 2.7. Statistical Analysis

Data are expressed as the mean SEM of three–four independent experiments. The data were processed using one-way ANOVA in GraphPad PRISM (GraphPad Software, La Jolla, CA, USA). Results with *P* < 0.05, 0.01, or 0.001 were considered statistically significant.

## 3. Results

### 3.1. Human PBMC Proliferation and Apoptosis in Response to Treatment with WT and *α*-Gal KO Bone Tissues

**Figure 2:**
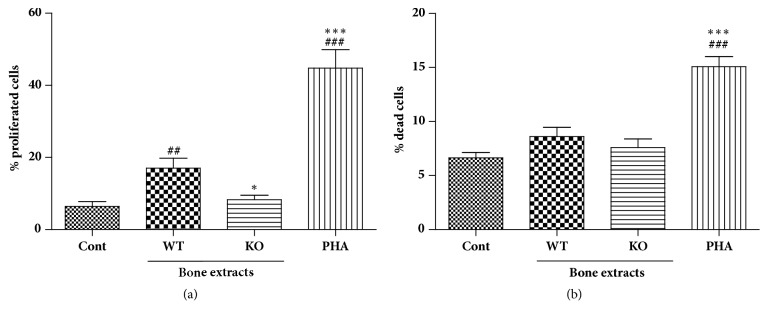
Effects on human PBMC proliferation. PBMCs were obtained from heparinized blood of healthy volunteers by histopaque gradient centrifugation. (a) PBMCs were labeled with CFSE and then bone extracts were added to PBMC cultures at 100 ug/ml concentration. Cells were incubated at 37C° for 72 hrs and analyzed on flow cytometry. (b) Cytotoxicity of bone extracts was evaluated with propidium iodide staining, followed by flow cytometry analysis. #*p* < 0.05, ##*p* < 0.005, and ###*p* < 0.001 indicate significant difference from the control group. *∗p* < 0.05, *∗∗p* < 0.005, and *∗∗∗p* < 0.001 indicate significant difference from the WT group. Experiments were repeated at least three times independently with hPBMC from 3-4 blood donors and bone extracts from 2-3 pigs. Data are expressed as means ± SEM.

To evaluate the immunogenicity of *α*-Gal KO bone tissues, we first treated human PBMCs with bone extracts prepared from WT or KO pigs and measured the extent of PBMC proliferation in response to the bone extracts. As shown in Figure 2(a), the WT extract induced significant PBMC proliferation; however, the percentage of proliferating cells in response to KO treatment was comparable to that of the untreated control cells. This effect was not due to cell death induced by the KO since treatment with KO bone extracts did not induce significant cell death compared to that with control or WT treatments (Figure 2(b)). Therefore, these results indicated that bone extracts from KO pigs do not stimulate proliferation of human PBMCs.

### 3.2. Inflammatory Cytokine Production by Human PBMCs in Response to Treatment with WT and *α*-Gal KO Bone Tissues

**Figure 3:**
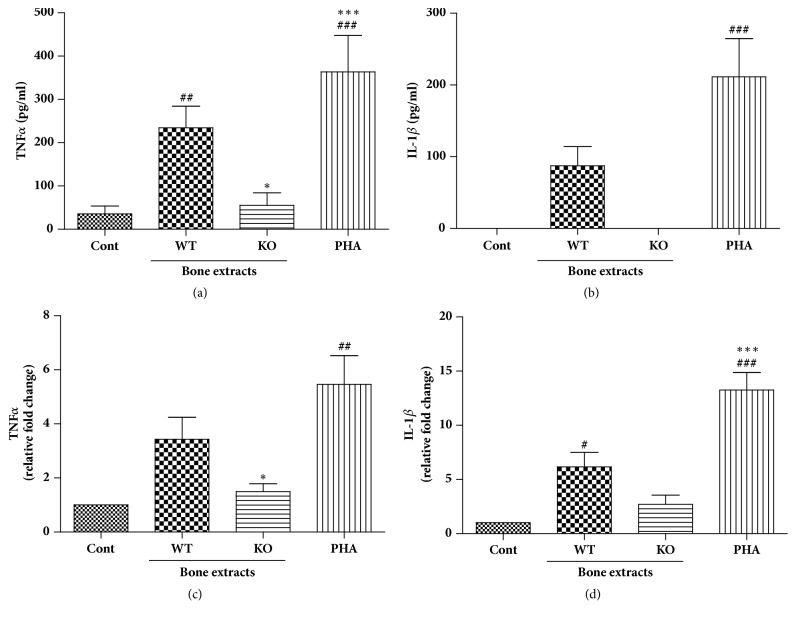
Effects of bone extracts on TNF-*α* and IL-1*β*production in human PBMC culture. PBMCs were obtained from heparinized blood of healthy volunteers by histopaque gradient centrifugation. Bone extracts were added to PBMC cultures at 100 ug/ml concentration and cells were incubated for 48 hrs for ELISA (a & b) or for 24 hrs for real time PCR (c & d). #*p* < 0.05, ##*p* < 0.005, and ###*p* < 0.001 indicate significant difference from the control group. *∗p* < 0.05, *∗∗p* < 0.005, and *∗∗∗p* < 0.001 indicate significant difference from the WT group. Experiments were repeated at least three times independently with hPBMC from 3-4 blood donors and bone extracts from 2-3 pigs. Data are expressed as means ± SEM. ND: not detected.

Immune cells produce various cytokines and chemokines when activated. Among these, proinflammatory cytokines such as TNF-*α* and IL-1*β* are secreted by activated macrophages/monocytes and cause tissue damage. Therefore, the protein and mRNA levels of these two cytokines were measured after treating human PBMCs with the bone extracts. Similar to the result obtained regarding proliferation of PBMCs, WT bone extracts stimulated secretion of TNF-*α* and IL-1*β* significantly. However, the levels of secreted TNF-*α* were lower when PBMCs were stimulated with the KO bone extract and the level of IL-1*β* was undetectable (Figures 3(a) and 3(b)). The mRNA levels of these cytokines were 2-3 fold lower in PBMCs stimulated with *α*-Gal KO pig bone extracts than that of PBMCs stimulated with WT bone extracts (Figures 3(c) and 3(d)). These results suggested that bone tissues without *α*-Gal induced weaker activation of human PBMCs.

### 3.3. CD4^+^ T Cell Activation in Response to Treatment with WT and *α*-Gal KO Bone Tissues

**Figure 4:**
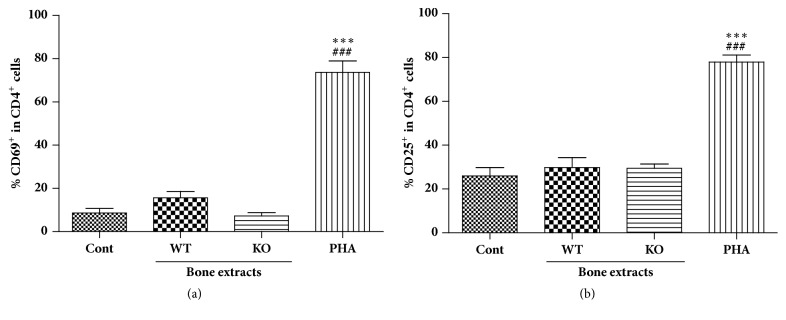
Effect of bone extracts on CD4+ T cell activation in human PBMC culture. PBMCs were obtained from heparinized blood of healthy volunteers by histopaque gradient centrifugation. Bone extracts were added to PBMC cultures at 100 ug/ml concentration cells were incubated for 24 hrs and then analyzed for CD69 (a) and CD25 (b) expression on the surface of CD4^+^ T cell* via* flow cytometry analysis. #*p* < 0.05, ##*p* < 0.005, and ###*p* < 0.001 indicate significant difference from the control group. *∗p* < 0.05, *∗∗p* < 0.005, and *∗∗∗p* < 0.001 indicate significant difference from the WT group. Experiments were repeated at least three times independently with hPBMC from 3-4 blood donors and bone extracts from 2-3 pigs. Data are expressed as means ± SEM.

Next, we examined the effect of treatment with *α*-Gal KO porcine bone tissue on CD4^+^ T cell activation. We analyzed CD69 and CD25 levels since CD69 is the early maker of T cell activation and CD25 is a high affinity receptor for a T cell growth factor (IL-2), which is expressed on the surface of activated T cells. As shown in Figure 4(a), 8.7% CD4^+^ T cells expressed CD69 in untreated control human PBMCs, whereas the percentage of CD4^+^ CD69^+^ T cells was higher (21%) in human PBMCs treated with WT bone extracts. Interestingly, when human PBMCs were treated with *α*-Gal KO bone extracts, CD4^+^ T cells were not activated and the percentage of CD4^+^ CD69^+^ cells was only 7.7%. There were no significant differences between WT and KO bone extract treatments for CD25 expression (Figure 4(b)).

**Figure 5:**
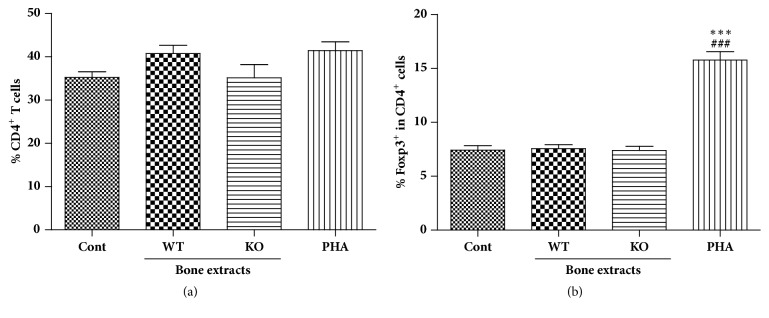
Effect of bone extracts on the percentage of CD4^+^ T cells and regulatory T cells. PBMCs were obtained from heparinized blood of healthy volunteers by histopaque gradient centrifugation. Bone extracts were added to PBMC cultures at 100 ug/ml concentration after 96 hrs, cells were stained for CD3 and CD4 using specific antibodies, and percentage of CD4^+^ T cells was assessed by flow cytometry analysis (a). Cells were further stained for intracellular expression of foxp3 and percentages of Tregs in CD4^+^ T cells were analyzed using flow cytometry analysis.. #*p* < 0.05, ##*p* < 0.005, and ###*p* < 0.001 indicate significant difference from the control group. *∗p* < 0.05, *∗∗p* < 0.005, and *∗∗∗p* < 0.001 indicate significant difference from the WT group. Experiments were repeated at least three times independently with hPBMC from 3-4 blood donors and bone extracts from 2-3 pigs. Data are expressed as means ± SEM.

After activation, CD4^+^ T cells proliferate and differentiate into certain types of CD4^+^ T cells such as regulatory T cells (Tregs). In previous experiments, proliferation of total human PBMCs was observed in cells treated with WT bone extracts (Figure 2(a)). However, when we examined the percentage of CD4^+^ T cells in human PBMCs, the levels were not meaningfully changed by treatment with either WT or KO bone extracts (Figure 5(a)). Foxp3 is a lineage marker of Tregs, a well-known CD4^+^ T cell subtype suppressing immune cell activation. The possibility that KO bone extracts may induce CD4^+^ T cell differentiation into Tregs was evaluated using Foxp3, and we found that bone extracts from pigs did not affect the percentage of Tregs in human PBMC regardless of *α*-Gal expression (Figure 5(b)).

### 3.4. T Cell Responses to Treatment with WT and *α*-Gal KO Bone Tissues

**Figure 6:**
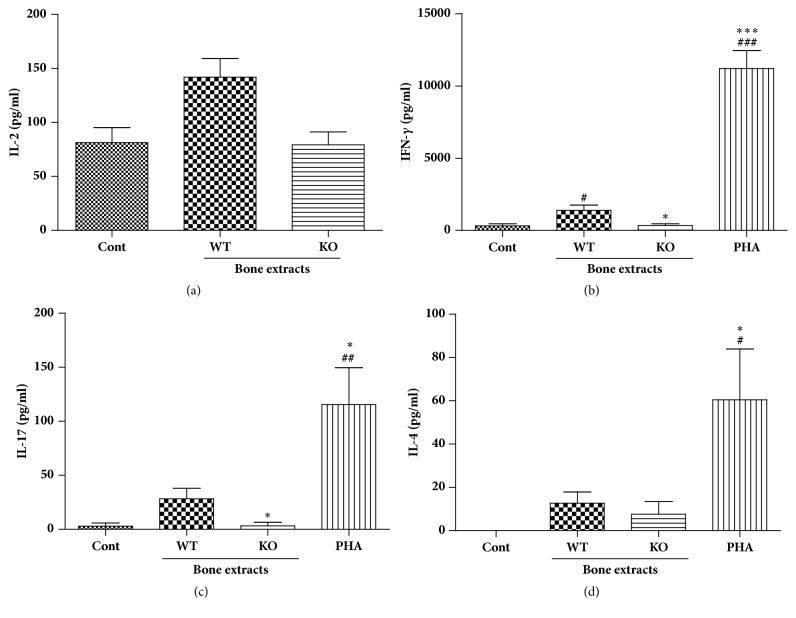
Effect of bone extracts on the cytokine expression in human PBMC. PBMCs were treated with 100 ug/ml of bone extracts for 96 hrs. Cell culture supernatants were prepared and used for cytokine ELISA such as IL-2 (a), IFN-*γ* (b), IL-17 (c), and IL-4 (d). #*p* < 0.05, ##*p* < 0.005, and ###*p* < 0.001 indicate significant difference from the control group. *∗p* < 0.05, *∗∗p* < 0.005, and *∗∗∗p* < 0.001 indicate significant difference from the WT group. Experiments were repeated at least three times independently with hPBMC from 3-4 blood donors and bone extracts from 2-3 pigs. Data are expressed as means ± SEM.

Activated T cells produce various cytokines that orchestrate adaptive immune responses. IL-2 is a growth factor for T cell proliferation and the level of this cytokine may closely correlate with T cell proliferation. The concentration of IL-2 was lower in KO bone extract-treated cells compared to that of WT bone extract-treated cells; however, the difference was not statistically significant (Figure 6(a)). IFN-*γ* is one of the signature cytokines involved in cell-mediated immune response, which is responsible for chronic rejection response to graft. As shown in Figure 6(b), IFN- *γ* levels increased in human PBMCs treated with WT bone extracts but were low in untreated control cells and human PBMCs treated with KO bone extracts. Similar result was obtained for IL-17 production as shown in Figure 6(c). IL-17 is a recently identified cytokine and is possibly involved in allograft rejection. IL-4 is produced mainly by CD4^+^ T cells and acts as negative regulator of CD4^+^ T cells producing either IFN-*γ* or IL-17. In this study, we found that the absence of *α*-Gal did not affect the production of IL-4 in response to pig bone extracts in human PBMCs (Figure 6(c)).

### 3.5. Activation of Signal Transduction Pathways in Response to Treatment with WT and *α*-Gal KO Bone Tissues

**Figure 7:**
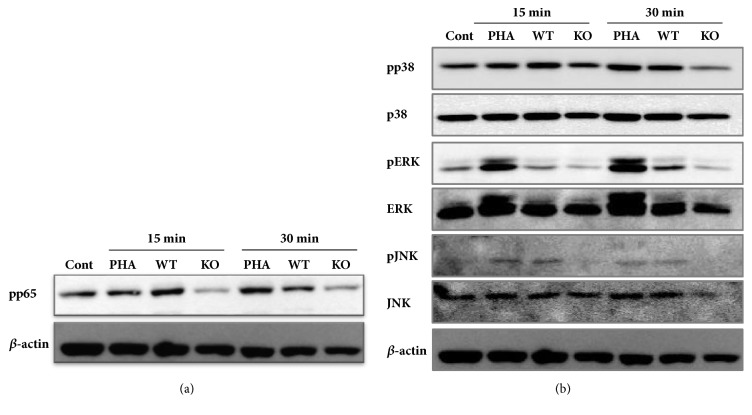
Effect of bone extracts on signal transduction in human PBMC culture. PBMCs were treated with bone extracts either from WT or from KO pigs for indicated time. Total or phosphoprotein levels of p65, JNK, ERK, and p38 in whole lysates were identified by specific antibodies* via* immunoblotting. Representative result is shown.

Finally, signal transduction pathways involved in cytokine production and immune cell activation were examined in human PBMCs treated either with WT or *α*-Gal KO bone extracts. NF-*κ*B and mitogen-activated protein kinase (MAPK) pathways are major players in inflammatory response. First, activation of NF-*κ*B was evaluated by determining the level of phosphorylation of the p65 subunit of NF-*κ*B. When human PBMCs were exposed to WT extracts, phosphorylation of p65 increased compared to that of the control. However, bone extracts from *α*-Gal KO did not induce activation of NF-*κ*B (Figure 7(a)). Next, we tested whether the deficiency of *α*-Gal expression in bone tissues affects the activation of MAPK pathways. As shown in Figure 7(b), phosphorylation of p38, ERK, and JNK increased in human PBMCs treated with WT bone extracts but not with *α*-Gal KO bone extracts. Taken together, these results indicate that reduced immune responses against bone tissue extracts from *α*-Gal KO are possibly due to lower levels of activation of signal transduction pathways such as NF-*κ*B, p38, ERK, and JNK.

## 4. Discussion

Xenotransplantation can resolve the chronic organ shortage for human organ transplantation, and pigs are considered appropriate sources for xenografts [5–8]. However, hyperacute rejection due to preexisting anti-*α*-Gal antibodies is a bottleneck. Hyperacute rejection of pig xenograft in humans can be prevented by knocking out the gene encoding *α*-galactosyltransferase in pigs as described in previous studies [6, 11]. However, acute and chronic rejections mediated mainly by T cells are inevitable barriers to successful xenotransplantation [21]. Therefore, reducing T cell responses against pig xenograft is the key strategy for avoiding acute or chronic rejection.

Previous studies demonstrated that aortic endothelial cells and mesenchymal stromal cells from *α*-Gal KO pigs triggered weaker human T cell responses* in vitro* [22]. Consistently, our results also showed that bone tissues from *α*-Gal KO pigs are less immunogenic and induced much weaker T cell responses as evidenced by lower levels of cell surface activation markers and cytokine production. Therefore, it is possible that *α*-Gal expression is involved in immune cell interaction and activation.

To further explore the possible mechanisms underlying reduced immunogenicity of *α*-Gal KO bone tissues, we investigated signal transduction pathways, such as the NF-*κ*B and MAPK pathways, which are responsible for immune cell activation and cytokine production [23, 24]. The transcription factor NF-*κ*B, highly expressed in the immune system, plays a leading role in the production of cytokines such as TNF-*α*, IL-1*β*, IL-6, and IFN-*γ* and immune cell activation. When this pathway is triggered, p65, a subunit of NF-*κ*B, is phosphorylated and translocated into the nucleus where it binds to promoters of cytokine-encoding genes [25, 26]. In the present study, we noted that NF-*κ*B activation was induced by bone tissue extracts of WT pigs but not by that of *α*-Gal KO pigs. In addition, MAPK pathway members such as ERK, p38, and JNK also participate in the transcription of various cytokine-encoding genes and mediate immune cell activation [24]. Our results suggest that the lack of *α*-Gal in bone tissues induces significantly lower phosphorylation levels of ERK1/2, JNK, and p38. Thus, our observations imply that *α*-Gal deficiency suppresses cytokine production and T cell responses via reduced signal transduction through the MAPK and NF-*κ*B pathway. The results showed that *α*-Gal KO pig bone xenografts may be used as scaffolds for the treatment of bone defects as an alternative to autografts and allografts. The current findings can be used to evaluate the compatibility of bone tissues from *α*-Gal KO pigs with human bone grafting as they provide sufficient and reliable evidence that warrant an extended trial and valid assessment.

## 5. Conclusion

We demonstrated a crucial role of *α*-Gal in the regulation of inflammatory cytokine production by human PBMCs and in T cell response against porcine bone tissues* in vitro*. The current findings enhance the understanding of the inflammatory mechanisms involved in the rejection of bone tissues from WT versus *α*-Gal KO pigs, and can be used to evaluate the compatibility of bone tissues from *α*-Gal KO pigs with human bone graft for the treatment of bone defects, thereby increasing the feasibility of clinical application in future.
